# Determining a new formula for calculating low-density lipoprotein cholesterol: data mining approach

**DOI:** 10.17179/excli2015-162

**Published:** 2015-03-26

**Authors:** Prabhop Dansethakul, Lalin Thapanathamchai, Sarawut Saichanma, Apilak Worachartcheewan, Phannee Pidetcha

**Affiliations:** 1 Excellence Service Center For Medical Technology and Quality Improvement, Faculty of Medical Technology, Mahidol University, Bangkok 10700, Thailand; 2 Center of Medical Laboratory Services, Faculty of Medical Technology, Mahidol University, Bangkok 10700, Thailand; 3 Division of Clinical Microscopy, Faculty of Medical Technology, Huachiew Chalermprakiet University, Samut Prakarn, Thailand; 4 Department of Clinical Chemistry, Faculty of Medical Technology, Mahidol University, Bangkok 10700, Thailand

**Keywords:** cholesterol, data mining, Friedewald formula, LDL-C, LDL-Cal, LDL-Direct, pace regression

## Abstract

Low-density lipoprotein cholesterol (LDL-C) is a risk factor of coronary heart diseases. The estimation of LDL-C (LDL-Cal) level was performed using Friedewald's equation for triglyceride (TG) level less than 400 mg/dL. Therefore, the aim of this study is to generate a new formula for LDL-Cal and validate the correlation coefficient between LDL-Cal and LDL-C directly measured (LDL-Direct). A data set of 1786 individuals receiving annual medical check-ups from the Faculty of Medical Technology, Mahidol University, Thailand in 2008 was used in this study. Lipid profiles including total cholesterol (TC), TG, high-density lipoprotein cholesterol (HDL-C) and LDL-C were determined using Roche/Hitachi modular system analyzer. The estimated LDL-C was obtained using Friedewald's equation and the homogenous enzymatic method. The level of TG was divided into 6 groups (TG<200, <300, <400, <500, <600 and < 1000 mg/dL) for constructing the LDL-Cal formula. The pace regression model was used to construct the candidate formula for the LDL-Cal and determine the correlation coefficient (*r*) with the LDL-Direct. The candidate LDL-Cal formula was generated for 6 groups of TG levels that displayed well correlation between LDL-Cal and LDL-Direct. Interestingly, The TG level was less than 1000 mg/dL, the regression model was able to generate the equation as shown as strong *r* of 0.9769 with LDL-Direct. Furthermore, external data set (n = 666) with TG measurement (36-1480 mg/dL) was used to validate new formula which displayed high* r* of 0.971 between LDL-Cal and LDL-direct. This study explored a new formula for LDL-Cal which exhibited higher *r* of 0.9769 and far beyond the limitation of TG more than 1000 mg/dL and potential used for estimating LDL-C in routine clinical laboratories.

## Introduction

The association between total cholesterol (TC) and the risk of developing atherosclerosis has been established by study from Framingham Heart Study (Kannel et al., 1971[8]). The recently of the National Cholesterol Education Program Adult Treatment Panel III (NCEP ATP III) guidelines focus on diagnosis and treatment effects on TC and low-density lipoprotein cholesterol (LDL-C). Therapy is target on lowering LDL-C value below a target value which depends on primary basis for treatment and appropriate the number patients' classification in risk categories values as compared with previous reports included the Friedewald formula (FF) and a direct homogenous assay (NCEP, 2001[10]). The FF is based on theoretical consideration which involved many factors and family history. The reference method for LDL-C concentration measurement which combined ultracentrifugation-precipitation is not practical for routine laboratory. So, a new generation of direct homogenous assays for LDL-C determination in serum has been developed with satisfactory degree of accuracy but it is expensive for using in developing countries (Bairaktari et al., 2005[2]; Nauck et al., 2002[9]).

Most clinical laboratory estimated LDL-C concentration in serum from FF with using TC, high-density lipoprotein cholesterol (HDL-C) and triglyceride (TG). TG is mainly from chylomicron and very-low-density lipoproteins (VLDL) assuming non HDL-C (TC-HDL-C) has little no change. However, TG level is too high, the LDL-C value is underestimated. This condition occurs in the postprandial condition or patient with normal non-HDL-C but high TG level. Now the LDL-C is used to manage for patients having risk of coronary heart disease and is a one marker for atherosclerosis (NCEP, 1994[11]; Cheng and Leiter, 2006[4]). Therefore, measurement of LDL-C has been required to estimate LDL-C values in clinical laboratories (NCEP, 1994[11]). Normally, the LDL-C in serum was calculated using FF based on used concentration of TC, TG and HDL-C (Friedewald et al., 1972[6]). However, LDL-Direct was determined using homogeneous enzymatic assays in case of non calculated LDL-C. The reliability of using FF was limited in TG concentration > 400 mg/dL that may be values of the LDL-C as underestimated (Chen et al., 2010[3]). Therefore, modified LDL-Cal formulas have been developed for estimate LDL-C to be appropriate for ethnic-specific as well as other population (Anandaraja et al., 2005[1]; Chen et al., 2010[3]; de Cordova and de Cordova, 2013[5]; Puavilai et al., 2009[13]; Vujovic et al., 2010[14]). The aim of this study is to investigate the candidate formula for LDL-Cal in TG < 1000 with validated the correlation coefficient (*r*) of the formula as compared with the FF and direct homogeneous assays.

## Material and Methods

### Sample population

A data set of 1786 individuals residing in urban Thailand was obtained from annual medical check-ups from the Center of Medical Laboratory Services of the Faculty of Medical Technology, Mahidol University in 2012 which was accreditation by ISO 15189 and participate external quality assessment with RIQAS^®^. Fasting blood sample of 12 hours overnight were analyzed in term of lipid profiles comprising of TC, TG, HDL-C, and LDL-C. All subjects were divided into six categories according to their TG value as 6 groups (A: TG< 200, B: < 300, C: < 400, D: < 500, E: < 600, and F: < 1000 mg/dL). 

### Lipid profiles measurements 

Lipid profiles measurement (low to high) composed of TC (107-413 mg/dL), TG (57-1000 mg/dL), HDL-C (19-119 mg/dL), and LDL-Direct (7-207.3 mg/dL) were determined by standard homogenous enzymatic method using automatic chemistry analyzer (Hitachi 911, Roche^®^). 

In general, the reported LDL-C was calculated using Friedewald formula from the following equation:


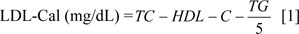


However, TG was greater than 400 mg/dL, the LDL-C was measured by direct LDL-Direct instead of LDL-Cal.

### Data mining analysis

The data mining analysis was analyzed using WEKA software, version 3.6.10 which is the collection of machine learning algorithms for data mining tasks (Hall et al., 2009[7]). The Pace regression which ones of data mining technique was approached to pattern relationship of explanatory LDL-Cal variables (TC, TG, HDL-C and LDL-Direct). It is a linear regression that showed to outperform other types of linear model-fitting methods, especially, in the cases of large and mutually dependent of variables in the data set (Wang, 2000[15]). The pace regression was used for constructed LDL-Cal equation. Correlation coefficient (*r*) was used to evaluate correlation between LDL-Cal and LDL-Direct.

### Statistical analysis

Statistical analysis was performed using SPSS Statistics 18.0 (SPSS Inc. USA). The six formulas for estimating LDL-C were performed using different equations (Anandaraja et al., 2005[1]; Chen et al., 2010[3]; de Cordova and de Cordova, 2013[5]; Friedewald et al., 1972[6]; Puavilai et al., 2009[13]; Vujovic et al., 2010[14]) and compared with our formula by observed *r *between LDL-Cal and LDL-Direct. In addition, validation of new formula was performed using new data set as called as external data set with difference of TG concentration (n = 666) in range of 36-1480 mg/dL composed of 551 individuals having TG < 400 mg/dL and 115 individuals having TG > 400 mg/dL as normal to abnormal level for calculating LDL-C.

## Results

**Figure 1:**
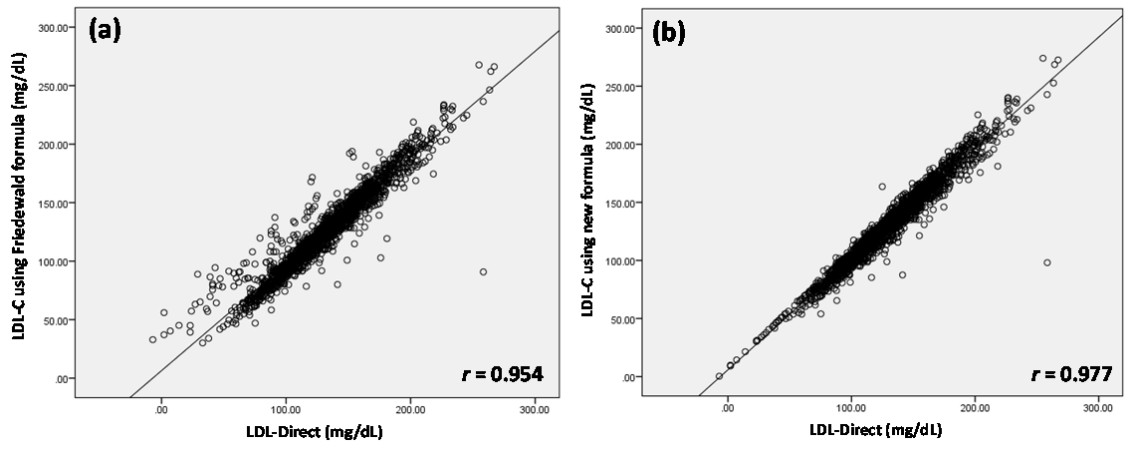
The comparative data and correlation coefficient (*r*) of LDL-C between LDL-Cal using Friedewald formula and LDL-Direct method (a) and LDL-Cal using new and LDL-Direct method (b)

**Figure 2:**
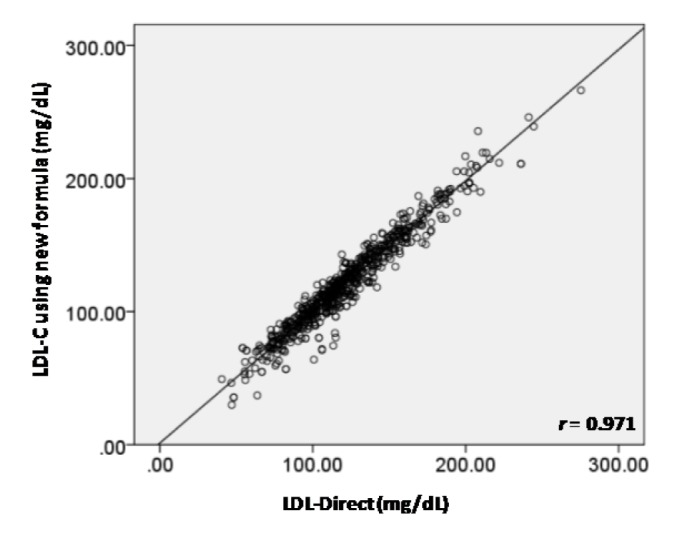
The comparative data and correlation coefficient *(r)* of LDL-C between LDL-Cal (using new formula)and LDL-Direct method for validation of new formula using external data set

The average values (mean ± SD) of TC, TG, and HDL-C were 213.16 ± 39.34, 139.76 ± 122.97 and 60.25 ± 15.97 mg/dL, respectively. Table 1(Tab. 1) shows the candidate LDL-Cal formula stratified by the levels of TG in groups A-F. It was found that *r* of six LDL-Cal formulas exhibited reliability *r* greater than 0.9759 compared with LDL-Direct. Interestingly, TG level of < 400, < 500, < 600 and < 1000 mg/dL displayed high *r* of 0.9792, 0.9759, 0.9759 and 0.9769, respectively. Furthermore, the other formulas (Anandaraja et al., 2005[1]; Chen et al., 2010[3]; de Cordova and de Cordova, 2013[5]; Friedewald et al., 1972[6]; Puavilai et al., 2009[13]; Vujovic et al., 2010[14]) were used to estimate LDL-C compared with LDL-Direct as shown in Table 2(Tab. 2). It observed that our candidate formula of LDL-C calculating as LDL-Cal = 0.995 (TC) - 0.9853 (HDL-C) - 0.1998 (TG) + 7.1449 provided the strong correlation (*r* = 0.9769) between direct measured LDL-Direct and LDL-C calculation than other formulas, particularly, compared with *r* of LDL-Cal by the original FF was 0.9540. Interestingly, TG < 400 mg/dL displayed *r *as 0.9792 greater than TG < 1,000, < 600, < 500, < 300 and < 200 mg/dL. However, in case of TG < 1000 mg/dL, *r* of 0.9769 was showed to be well correlation between LDL-Cal and LDL-Direct. Figure 1a(Fig. 1) displayed the comparative data and *r* (0.954) of LDL-C between LDL-Cal using FF and LDL-Direct method. Furthermore, comparative data and *r* (0.977) of LDL-C with calculated from the new formula (groups A-F) in this study and LDL-Direct method was shown in Figure 1b(Fig. 1). It exhibited good correlation coefficient between LDL-Cal (using FF and new formula) and LDL-Direct (Figures 1a and b(Fig. 1)). Additionally, confirmation or validation of new formula were evaluation using external data set (n = 666) with measurement of TG level from normal to high level (36-1480 mg/dL) for calculating LDL-C which compared with LDL-Direct measurement. It exhibited high *r* of 0.971 between LDL-Cal (using new formula) and LDL-Direct method as shown in Figure 2(Fig. 2).

## Discussion

The present study demonstrated the candidated LDL-Cal formula as used as in routine clinical laboratories. The original FF (Table 2(Tab. 2)) displayed *r* of 0.954 that compared with LDL-Direct. Although, FF is limited to TG < 400 mg/dL (Friedewald et al., 1972[6]) but in our study, the FF can be used to estimate LDL-C value in TG< 1000 mg/mL. The five different formulas were used to estimate LDL-C composed of Chen's formula (2010[3]), Anandaraja's formula (2005[1]), Puavilai's formula (2009[13]), Vujovic's formular (2010[14]) and de Cordova's formula (2013[5]) as shown in Table 2(Tab. 2), Considering our formula for estimated LDL-C, it exhibited *r* of 0.977 outperformed all LDL-Cal formulas. Chen et al. (2010[3]) estimated LDL-C using LDL-Cal = non-HDL-C×90 %-TG ×100 % to calculate LDL-C (n = 2180) in Chinese population. The *r* between LDL-Cal and LDL-Direct was 0.723 that well correlated with LDL-Direct in TG > 400 mg/dL as well as validated in other populations (Nigam, 2014[12]). The Anandaraja's formula (LDL-Cal = 0.9TC -0.9TG/5-28) was studied in Indian population (n = 1000) that *r* of 0.88 correlated between LDL-Direct and LDL-Cal (2005[1]). But removed TG > 350 mg/dL, the *r* was increased to 0.92, however, this formula was documented as not better than FF for a different Indian study (Nigam, 2014[12]). Puavilai et al. (2009[13]) used modified FF (LDL-Cal = TC-HDL-C-TG/6) to calculate LDL-C (n = 999) in Thai population. It was found that the *r* between LDL-Direct and LDL-Cal were 0.884 when TG level was less than 300 mg/dL. The simple formula of LDL-Cal = ¾ (TC-HDL-C) was provided by de Cordova and de Cordova (2013[5]) as used 10664 subjects. It was high correlation with LDL-Direct (*r* = 0.93), but this formula was not better than FF in healthy South African population and other population (Nigam, 2014[12]). In addition, Vujovic et al. (2010[14]) used LDL-Cal = TC-HDL-C-TG/3 for estimated LDL-C (*n* = 1010) in Serbian population. It was found that *r* was displayed of 0.96 compared with LDL-Direct. However, it was not validated in serum with TG > 400 mg/dL (Nigam, 2014[12]). As the results from five LDL-Cal formula (Anandaraja et al., 2005[1]; de Cordova and de Cordova, 2013[5]; Friedewald et al., 1972[6]; Puavilai et al., 2009[13]; Vujovic et al., 2010[14]), it was limited for TG < 400 mg/dL, except, Chen's formula (2010[3]) found *r* of 0.723 for TG > 400mg/dL. In our study, the LDL-Cal = 0.9955TC - 0.9853HDL-C - 0.1998TG + 7.1449 displayed well correlation between LDL-Direct and LDL-Cal that showed the best correlation when compared with other formula to estimate LDL-C (Table 2(Tab. 2)). Moreover, TG < 400 mg/dL displayed high* r *of 0.9792 than previous reported by other LDL-C formula (Anandaraja et al., 2005[1]; Chen et al., 2010[3]; de Cordova and de Cordova, 2013[5]; Friedewald et al., 1972[6]; Puavilai et al., 2009[13]; Vujovic et al., 2010[14]).

In conclusion, this finding is anticipated to validate a new formal for estimating LDL-C as shown the strongest correlated with direct measured LDL-C and beyond the limitation of TG up to 1000 mg/dL. It could be potential used for estimating LDL-C in routine clinical laboratories.

## Acknowledgements

We thank the Center of Medical Laboratory Services of the Faculty of Medical Technology, Mahidol University for measuring blood chemistry and the data set used in this study.

## Figures and Tables

**Table 1:**
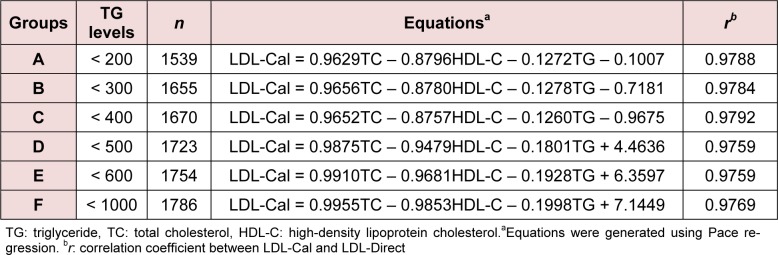
The candidate formula of estimated LDL-C (LDL-Cal) with concentration of triglyceride levels

**Table 2:**
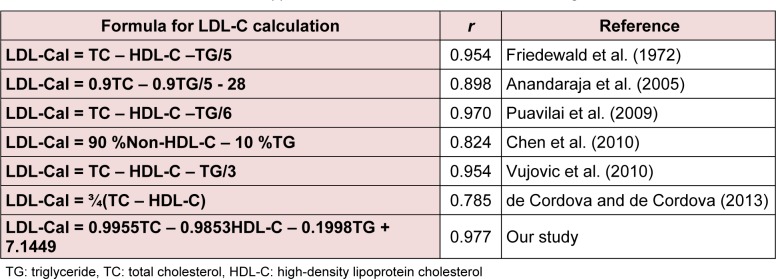
The correlation coefficient (*r*) between LDL-Cal and LDL-Direct using different formulas
